# A Precisely Flow-Controlled Microfluidic System for Enhanced Pre-Osteoblastic Cell Response for Bone Tissue Engineering

**DOI:** 10.3390/bioengineering5030066

**Published:** 2018-08-12

**Authors:** Eleftheria Babaliari, George Petekidis, Maria Chatzinikolaidou

**Affiliations:** 1 Department of Materials Science and Technology, University of Crete, Crete 70013, Greece; ebabaliari@iesl.forth.gr (E.B.); georgp@iesl.forth.gr (G.P.); 2 Foundation for Research and Technology-Hellas (FORTH), Institute of Electronic Structure and Laser (IESL), Crete 70013, Greece

**Keywords:** microfluidics, collagen, osteogenic differentiation, cell orientation, MC3T3-E1 pre-osteoblasts

## Abstract

Bone tissue engineering provides advanced solutions to overcome the limitations of currently used therapies for bone reconstruction. Dynamic culturing of cell-biomaterial constructs positively affects the cell proliferation and differentiation. In this study, we present a precisely flow-controlled microfluidic system employed for the investigation of bone-forming cell responses cultured on fibrous collagen matrices by applying two flow rates, 30 and 50 μL/min. We characterized the collagen substrates morphologically by means of scanning electron microscopy, investigated their viscoelastic properties, and evaluated the orientation, proliferation and osteogenic differentiation capacity of pre-osteoblastic cells cultured on them. The cells are oriented along the direction of the flow at both rates, in contrast to a random orientation observed under static culture conditions. The proliferation of cells after 7 days in culture was increased at both flow rates, with the flow rate of 50 μL/min indicating a significant increase compared to the static culture. The alkaline phosphatase activity after 7 days increased at both flow rates, with the rate of 30 μL/min indicating a significant enhancement compared to static conditions. Our results demonstrate that precisely flow-controlled microfluidic cell culture provides tunable control of the cell microenvironment that directs cellular activities involved in bone regeneration.

## 1. Introduction

In recent years, demand for bone grafts across the population has been strong and increasing. However, the limited volume of bone grafts and the frequent morbidity of patients associated with current therapies lead to the need for the development of advanced therapeutic strategies for bone regeneration. Tissue engineering and biofabrication have both emerged as promising fields for the development of new bone graft substitutes to avoid the limitations of the current grafts [1]. Besides the selection of the appropriate biomaterial and the cell source for a successful tissue-engineered bone reconstruction, several other issues are important, such as the optimization of the culturing system.

Cells in a multicellular organism live in a considerably different environment compared to conventional static cultures. They are attached to softer materials than the glass and plastic substrates on which most studies are done in vitro, and are surrounded by fluid and nutrients. Significant evidence shows that physical parameters such as fluid shear, mechanical forces, flexibility and media accessibility can have profound effects on cell growth and differentiation [2]. Dynamic culturing of cell-biomaterial constructs has been reported to have a positive effect on cell proliferation [3,4] and cell differentiation [5]. Moreover, it has been shown that dynamic culture conditions enhance the proliferation and differentiation of bone forming cells [6,7]. Bancroft et al. [8] cultured marrow stromal osteoblasts on a titanium fiber mesh under flow conditions with different flow rates. With all flow rates, alkaline phosphatase (ALP) activity was dramatically increased compared to static cultures. Additionally, the total calcium content of the cultured scaffolds increased with increasing flow rate. Furthermore, Gomes et al. [6] studied the effect of flow perfusion on the osteogenic differentiation of bone marrow stromal cells on scaffolds made from a blend of starch with ethylene vinyl alcohol, and a blend of starch with polycaprolactone. The proliferation and ALP activity were similar for both scaffold types and both culturing conditions. However, the calcium deposition was enhanced on both types of scaffolds under flow. Holtorf et al. [9] cultured rat bone marrow stromal cells on titanium fiber mesh scaffolds and examined the ability of cells to differentiate into osteoblasts under flow perfusion with/without the use of dexamethasone. They observed that under flow without the use of dexamethasone, cellularity, ALP activity, calcium deposition and osteopontin secretion were enhanced. A further enhancement of osteogenic differentiation was observed when dexamethasone was present. Moreover, Leclerc et al. [7] fabricated polydimethylsiloxane (PDMS) microdevices with a three-dimensional (3D) microstructured channel network where they cultured mouse calvarial osteoblastic cells MC3T3-E1 under static and flow conditions. The ALP activity was enhanced 3- and 7.5-fold within the microdevices under static and flow conditions compared to static cultures in PDMS-coated surfaces. These results implied the importance of the biomaterial in combination with the flow perfusion on osteogenic differentiation. Considering this approach crucial for tissue maturation, and taking into account the dynamic in vivo conditions in the human body, we focused on setting a precisely flow-controlled microfluidic system, which, together with the development of a fibrous biomaterial matrix for the cells, would be appropriate for the investigation of dynamic cell culture experiments.

Microfluidic cell cultures reflect more appropriately the in vivo environment of cells in tissues such as the normal fluid flow within the body, consistent nutrient delivery, effective waste removal and mechanical stimulation due to fluid shear forces [10]. It is recognized that fluid flow exerts a shear stress on adherent cells, introducing one of the strongest stimuli in the responses of bone cells [11]. It provides sufficient levels of oxygen and nutrients within the biomaterial matrix as well as mechanical stimulation to the cells [12]. Laminar flow regimes, small length scales, and diffusion dominated mass transport characterize the microfluidic devices. These features can be used to provide a biomimetic environment for cell cultures, and consequently advance conventional static cultures by presenting conditions that resemble the intracellular environment [10,12]. Moreover, small volumes of media in microfluidic cell culture devices reflect more appropriately the physiological condition of cells in tissues than cells cultured in larger volumes, due to faster consumption of nutrients and increased concentration of metabolites and secreted products, similar to densely packed tissues [10].

Both gelatin and collagen are natural biomaterials, biocompatible, biodegradable, easily available and highly versatile. Collagen is the major component of the bone matrix and makes up approximately 30% of all body proteins [13]**,** while gelatin is an irreversibly hydrolyzed form of collagen with a chemical composition closely similar to that of its parent collagen. Cells cultured onto collagen hydrogels are surrounded by native extracellular matrix (ECM) proteins and can remodel their fibrous matrix. This remodeling process is controlled by local and global physical cues, resulting in changes of the mechanical characteristics. This dynamic crosstalk between cells and the matrix resembles the situation occurring in vivo [14]. Remodeling of the ECM network is crucial in tissue development, fibrosis, and functional tissue engineering, and presents an advantage of using the collagen hydrogel model system [15]. 

It has been shown that the mechanical properties of the substrate produce a profound impact on cell and tissue behavior [16]. When cells adhere to a substrate with adhesion molecules on their cell membrane, such as integrins, the cytoskeleton transmits traction forces between the multiple adhesion points, deforming the culture substrate according to its stiffness. Thus, cells can feel the stiffness of their substrates and respond by reorganizing their cytoskeleton and altering the expression of adhesion molecules [17]. Cell-cell adhesion and cell-substrate adhesion are important interactions that modulate intracellular signaling pathways for proliferation and differentiation, as well as cellular events like cell locomotion and gene expression [18]. Thus, it is useful to characterize the mechanical features of the substrates to have a better insight into the mechanical environment of the cells, and to evaluate the cell-mediated remodeling together with the resulting changes in the functional mechanical properties of the tissue-forming material.

In this study, we first used gelatin as a model test substrate to investigate the potential of pre-osteoblastic cells for orientation under flow conditions in the designed precisely flow-controlled microfluidic system, as well as their ability to form extracellular matrix as indicated by the collagen secretion. Once receiving promising results on the pre-osteoblastic cell response on gelatin substrates in the microfluidic chamber, we focused our investigation on the cell response on fibrous collagen substrates. For this, we characterized the collagen hydrogels morphologically by means of scanning electron microscopy (SEM), investigated their viscoelastic properties, and evaluated the orientation, proliferation and osteogenic differentiation capacity of pre-osteoblastic cells cultured on them.

## 2. Materials and Methods

### 2.1. Design of the Microfluidic System

**Figure 1:**
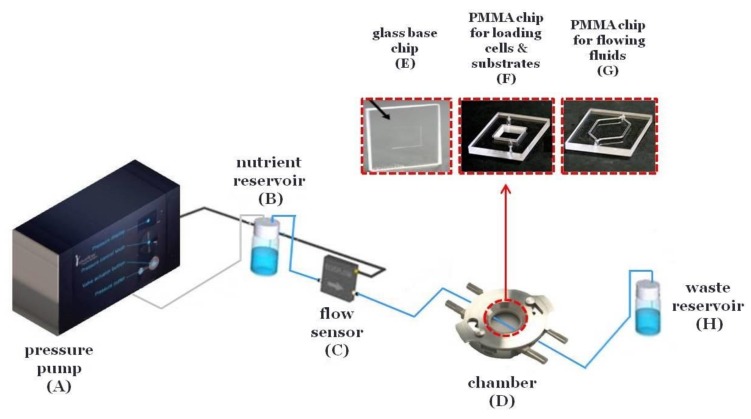
Illustration of the microfluidic system with precisely controlled flow. The microfluidic system is composed of a pressure pump (**A**) connected with a nutrient reservoir (**B**), a precisely controlled flow sensor (**C**), a chamber (**D**) including the microfluidic devices (**E**, **F** or **G**) and a waste reservoir (**H**).

The microfluidic system is composed of a pressure pump (Elveflow) (Figure 1A) connected with a precisely controlled flow sensor (Elveflow) (Figure 1C) and a chamber (Dolomite) (Figure 1D) including the microfluidic devices (Dolomite). The microfluidic devices are composed of a glass base chip for the cell layer and substrate (Figure 1E) and poly(methyl methacrylate) (PMMA) chips with PDMS gaskets for loading cells and substrates (10 mm × 10 mm × 3 mm) (Figure 1F) and for flowing fluids across the cells and substrates (20.4 mm × 11 mm × 100 μm) (Figure 1G). Briefly, silicon tubing connects the pressure pump (Figure 1A) with a reservoir containing the nutrient (Figure 1B). Then, 0.5 mm interior diameter poly(tetrafluoroethylene) (PTFE) tubing connects the reservoir (Figure 1B) with the flow sensor (Figure 1C) and finally with the chamber (Figure 1D) and the waste reservoir (Figure 1H). Both the chamber (Figure 1D) and the waste reservoir (Figure 1H) were inside a 5% carbon dioxide (CO_2_) incubator at 37 °C.

### 2.2. Preparation of Gelatin Substrates

A 2% *w*/*v* gelatin solution [gelatin from bovine skin, Type B (Sigma-Aldrich, St. Louis, MO, USA) was dissolved in water] was used for coating the glass base chip of the microfluidic system and dried for 2 h before seeding the cells.

### 2.3. Preparation of Collagen Substrate

For the preparation of a 3 mg/mL collagen gel, a 6 mg/mL collagen stock solution [collagen type I, rat tail (Sigma-Aldrich, St. Louis, MO, USA) was dissolved in 0.02 M acetic acid in water] was mixed with 10× phosphate buffer saline (PBS) (Sigma-Aldrich, St. Louis, MO, USA) and water, followed by neutralization with sodium hydroxide (NaOH) 0.5 M. Then, the 3 mg/mL collagen solution was used for the chip coating. Gelation was performed by incubating the collagen solution for 48 h at 4 °C for the formation of thick fibers. Thereafter, collagen gel dried for 3 h, washed with water and 1× PBS (Sigma-Aldrich, St. Louis, MO, USA) and was completely immersed with culture medium overnight in a 5% CO_2_ incubator at 37 °C before seeding the cells.

### 2.4. Characterization of the Collagen Substrates

#### 2.4.1. Characterization of Collagen Substrates by SEM

The collagen fibrous material substrates were dried for 3 h, sputter-coated with a gold layer of 20 nm thickness (Baltec SCD 050, BAL-TEC AG, Balzers, Liechtenstein) and observed by placing them in the sample holder in a perpendicular position under a scanning electron microscope (JEOL JSM-6390 LV, Jeol USA Inc, MA, USA) with an accelerating voltage of 15 kV. The images captured show cross-sections of the collagen fibrous material substrates on the glass substrates. 

#### 2.4.2. Rheological Characterization of Collagen Substrates

An Anton Paar MCR 501 (Anton Paar GmbH, Graz, Austria) stress-controlled rheometer was used for all measurements. In order to conduct rheological measurements with homogenous strain field while avoiding slippage, we used homemade serrated cone-plate geometry (cone angle = 3.22°, diameter = 25 mm), appropriately calibrated and tested with similar soft matter samples. Moreover, to minimize solvent (water) evaporation, we utilized a homemade solvent trap, which completely seals the sample from the environment and creates a saturated water vapor atmosphere. Measurements were performed at 37 °C following a well-defined experimental protocol to ensure reproducibility. The protocol involves dynamic strain sweep tests at a given frequency (1 rad/s) to determine the extent (maximum strain amplitude) of the linear regime of the materials and subsequently, Dynamic Frequency Sweep (DFS) tests at low strain amplitude in the linear regime (typically <1%) to measure the linear viscoelastic response of the sample at a range of frequencies (typically 0.1 to 100 rad/s).

### 2.5. Pre-Osteoblastic Cell Culture Maintenance

MC3T3-E1 osteoblast-like cells from newborn mouse calvaria are a non-transformed cell line that exhibits an osteoblastic phenotype. The cells used in this study were obtained from DSMZ GmbH (Braunschweig, Germany) (DSZM no: ACC 210) and have been described to differentiate to osteoblasts and produce type I collagen [19]. Cells were grown in cell culture flasks using culture medium [alpha-MEM (Sigma-Aldrich, St. Louis, MO, USA), supplemented with 10% Fetal Bovine Serum (FBS) (Sigma-Aldrich, St. Louis, MO, USA), 2 mM glutamine (Sigma-Aldrich, St. Louis, MO, USA), 50 IU/mL penicillin (Sigma-Aldrich, St. Louis, MO, USA), and 50 g/mL streptomycin (Sigma-Aldrich, St. Louis, MO, USA)] in a 5% CO_2_ incubator (Thermo Scientific or Heal Force) at 37 °C. Confluent cells were washed with 1× PBS (Sigma-Aldrich, St. Louis, MO, USA) and passaged after trypsinization [0.25% trypsin in 1 mM ethylenediaminetetraacetic acid (EDTA) (Gibco, Thermo Fisher Scientific, Waltham, MA, USA)], seeded at 80% confluence and cultured for 5 days before the next passage [20]. For the cell differentiation experiments, cells were cultured in osteogenic medium [culture medium supplemented with 50 μg/mL ascorbic acid (Sigma-Aldrich, St. Louis, MO, USA) and 10 mM β-glycerophosphate (Sigma-Aldrich, St. Louis, MO, USA)], which initiates a process directing cells into an osteoblastic differentiation pathway [21]. 

### 2.6. Static and Dynamic Cell Cultures

8 × 10^4^ cells/cm^2^ were cultured on the glass base chip of the microfluidic system, which was coated either with the gelatin film or the collagen substrate and kept at rest overnight in a 5% CO_2_ incubator at 37 °C. The next day, the perfusion of the culture was started and completed in the CO_2_ incubator. The osteogenic medium in the reservoir was changed every 3 days. Flow rates of 30 and 50 μL/min were used. The flow rates that we used, and consequently the speed, are similar to those of the small arteries in the bloodstream (arterioles, capillaries, and venules). Table 1 and Table 2 show the flow rates and speed in the blood circulation and in the microfluidic system, respectively.

The shear stress, *σ*, exerted on the cells is predicted by the equation [24]:

*σ* = (6η*Q*)/(bh^2^)

where η is the viscosity of the osteogenic medium (η = 0.01078 g·cm^−1^·s^−1^), *Q* is the volumetric flow rate, b is the width of the PMMA chip with PDMS gasket for flowing fluids across the cells and substrates (b = 11 mm) and h is the height of gasket (h = 100 μm). Table 3 shows the flow rates and the shear stresses in the microfluidic system.

For comparative purposes, 8 × 10^4^ cells/cm^2^ were cultured under static conditions, both in the chamber and in conventional static cultures, which were coated with gelatin film or collagen substrate. Finally, the osteogenic medium was changed every 3 days. All cell culture experiments were performed using glass substrates as control. However, the term “glass” refers to the static culture on a round glass substrate placed inside a well plate, while “microdevice glass” refers to the static culture, but inside the chamber.

### 2.7. Pre-Osteoblastic Cell Orientation by Optical Microscopy

MC3T3-E1 cells were visualized the first, fourth and seventh day by optical microscopy using a Zeiss Axiovert 200 microscope (Carl Zeiss, Oberkochen, Germany). Images were taken by a ProgRes^®^ CFscan Jenoptik camera (Jenoptik, Jena, Germany) using the ProgRes^®^ CapturePro 2.0 software and an objective lens at a 10-fold magnification. 

To investigate changes in directional orientation of MC3T3-E1 cells under static and flow conditions, the “Local gradient orientation” for directionality was performed using the Fiji ImageJ plug-in “Directionality” [25]. On top of the histogram, the plug-in generates statistics on the highest peak observed, which is fitted by a Gaussian function considering the periodic nature of the histogram. In the tables, the “Direction (°)” column reports the center of the Gaussian; the “Dispersion (°)” column refers to the standard deviation of the Gaussian; the “Amount” column presents the sum of the histogram from center−std to center+std, divided by the total sum of the histogram; the “Goodness” column indicates the goodness of the fit, where 1 is good, 0 is bad. 

### 2.8. Cell Morphology by SEM

2 × 10^4^ MC3T3-E1 cells in culture medium were seeded on collagen fiber networks and placed in the CO_2_ incubator at 37 °C for one day. Then, the samples were removed from the incubator, washed twice with 0.1 M sodium cacodylate buffer (SCB) and fixed with 2% glutaraldehyde (GDA) and 2% paraformaldehyde (PFA) in 0.1 M SCB for 30 min. Thereafter, they were washed twice with 0.1 M SCB and dehydrated in increasing ethanol concentrations from 30–100%. Finally, the samples were dried in a critical point drier (Baltec CPD 030, BAL-TEC AG, Balzers, Liechtenstein), sputter-coated with a gold layer of 20 nm thickness (Baltec SCD 050, BAL-TEC AG, Balzers, Liechtenstein), and observed under a scanning electron microscope (JEOL JSM-6390 LV, Jeol USA Inc, MA, USA) with an accelerating voltage of 15 kV [20].

### 2.9. Pre-Osteoblastic Cell Proliferation by Total Protein

The total protein concentration was determined using the Bradford reagent (Sigma-Aldrich, St. Louis, MO, USA). The Bradford assay depends upon the change in absorbance based on the proportional binding of the dye Coomassie Brilliant Blue to proteins. Cells were harvested from the chamber or from the static culture by trypsinization on day seven. Then, they were placed in 1.5 mL eppendorf tubes, collected by centrifugation at 4000 rpm for 15 min and washed with 1 mL 1× PBS. Afterward, a solution of 100 μL lysis buffer [0.1% Triton X-100 (Sigma-Aldrich, St. Louis, MO, USA) in Tris/HCl, pH 10] was added in each tube and they were incubated overnight in a freezer at −80 °C. The next day, cells were thawed. After thawing, aliquots of lysed cells were used to determine the protein concentration and alkaline phosphatase (ALP) activity (as described in Section 2.11). For the assays, 96-well plates were used. Briefly, 5 μL lysate or 5 μL lysis buffer (as blank), 15 μL Tris/HCl, pH 10, and 200 μL Coomasie Blue G-250 were added in each well of the 96-well plate and were incubated for 5 min at room temperature. The absorbance was then measured in a spectrophotometer at a wavelength of 595 nm and the total protein concentration was determined by means of a calibration curve. For each experiment, three replicates were used.

### 2.10. Determination of the Produced Extracellular Collagen

A modified Sirius red assay was used in order to stain the collagen produced in the extracellular matrix [26]. Sirius red F3B is an elongated molecule containing six sulphonic acid groups. Collagen is a basic protein and it is thus likely that the sulphonic groups of the dye may interact at low pH with the amino groups of lysine and hydroxylysine, and with the guanidine groups of arginine [27]. On the seventh day of the differentiation experiments, the supernatants were collected and stored at −80 °C for collagen determination. Briefly, 75 μL of water was added into 25 μL of supernatant or plain culture medium (blank) into 2 mL eppendorf tubes. Then, 1 mL of dye solution [0.1 gr Sirius red F3B (Sigma-Aldrich, St. Louis, MO, USA) dissolved in 100 mL acetic acid 0.5 M] was added and the samples were incubated at room temperature for 30 min. Afterward, the samples were centrifuged at 15,000 G for 15 min to pellet the collagen–dye complex and washed with 0.5 mL of 0.1 M hydrochloric acid (HCl) to remove the unbound dye. The stained material was dissolved in 0.5 mL of 0.5 M NaOH, and 200 μL aliquots were transferred into 96-well plates. The absorbance was measured in a spectrophotometer at a wavelength of 530 nm. The collagen was determined by means of a calibration curve that was created by staining known concentrations of collagen type I with the Sirius red assay. Samples were analyzed in triplicates. 

### 2.11. Alkaline Phosphatase (ALP) Activity and Normalization by the Total Protein

The levels of the alkaline phosphatase activity that indicate osteoblast differentiation were determined by means of an enzymatic activity assay. On the seventh day, cells were harvested from the chamber or from the static culture by trypsinization. Cell lysates were used for measurement of ALP activity in 96-well plates. In each well, 95 μL lysate or 95 μL lysis buffer (as blank), 5 μL Tris/HCl pH 10 and 100 μL of 2 mg/mL p-nitrophenylphosphate (pNNP) substrate [2 mg/mL pNNP substrate (Sigma-Aldrich, St. Louis, MO, USA) solution in 50 mM Tris/HCl pH 10] were added and the plate was incubated for 60 min at 37 °C. The absorbance was measured in a spectrophotometer (Molecular Devices SpectraMax M2 (San Jose, CA, USA) or SYNERGY HTX (BioTek, Winooski, VT, USA) at a wavelength of 405 nm. The alkaline phosphatase activity was determined by means of a calibration curve with known concentrations of p-nitrophenol. ALP activity was calculated using the equation units = nmol p-nitrophenol/min [28], normalized with total protein concentration and expressed as specific activity. Samples were analyzed in triplicates. 

### 2.12. Statistical Analysis

Cellular proliferation, collagen, alkaline phosphatase results are presented as mean values ± standard error to the mean (SEM). Statistical analysis was performed with the student’s *t*-test (GraphPad Prism 5 software, GraphPad software, San Diego, CA, USA). A *p value of < 0.05 was considered significant.

## 3. Results

### 3.1. Pre-Osteoblastic Cell Morphology and Orientation on Gelatin Substrates under Static and Flow Conditions

**Figure 2:**
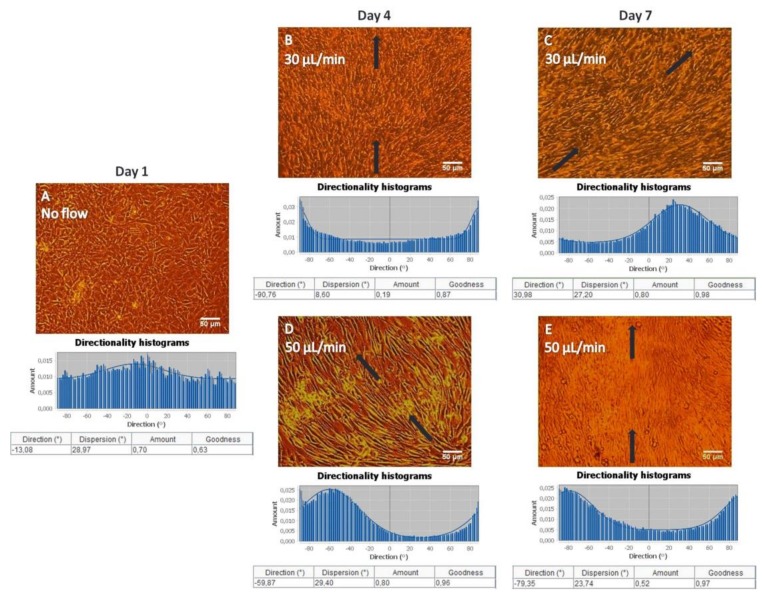
Morphology of MC3T3-E1 cells inside the flow perfusion culture system using gelatin substrates under static conditions (**A**), and under flow conditions applying 30 (**B**,**C**), and 50 μL/min (**D**,**E**) visualized by optical microscopy (ten-fold magnification, the scale bars represent 50 μm). The black arrows represent the direction of the flow. Directionality histograms and tables with the statistics generated by means of the Fiji ImageJ plug-in “Directionality” [25] are presented directly under the optical microscopy images (**A**–**E**).

Figure 2 illustrates the pre-osteoblastic cell morphology on a gelatin substrate under static (Figure 2A) and flow conditions, flow rates of 30 (Figure 2B,C) and 50 (Figure 2D,E) μL/min, at three different time points (1 day, 4 days, 7 days). We observed that the MC3T3-E1 cells attached well, exhibiting a spindle-like elongated shape and filopodia [19], and proliferated well both under static and flow conditions. Interestingly, under flow conditions, cells appeared oriented along the direction of flow, whereas cells depicted a random orientation under static conditions. This is clearly shown in Figure 2, where black arrows represent the direction of flow. Additionally, this is evidenced by the directionality histograms below the optical microscopy images, where the amount is clearly concentrated each time at the direction of flow [~−90° (Figure 2B), ~30° (Figure 2C), ~−60° (Figure 2D) and ~−80° (Figure 2E)] compared to the static culture (Figure 2A). 

### 3.2. Collagen Production of MC3T3-E1 Cells on Gelatin Films under Static and Flow Conditions

**Figure 3:**
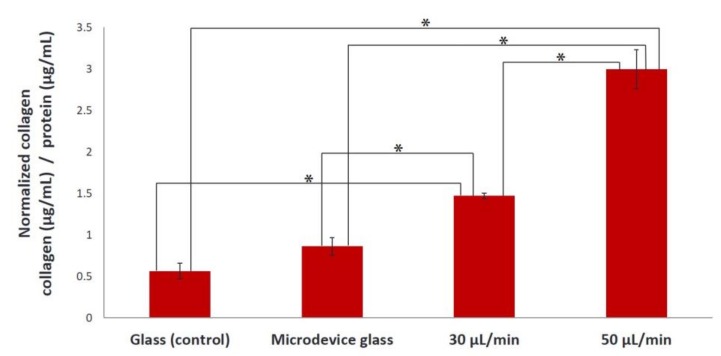
Normalized levels of collagen secreted by MC3T3-E1 cells on gelatin substrates after 7 days of culture under static conditions (glass and microdevice glass) and under flow conditions (flow rates of 30 and 50 μL/min). A *p value of < 0.05 was considered significant.

Figure 3 illustrates the normalized levels of collagen (collagen/protein) produced in the extracellular matrix on day 7 under static and flow conditions when gelatin film was used as a substrate. We observed that the collagen secreted by MC3T3-E1 cells under the flow rate of 30 μL/min was significantly higher compared to static conditions (glass and microdevice glass) (*p < 0.01), with a remarkably significant increase when applying the flow rate of 50 μL/min (*p < 0.01). Specifically, the collagen produced in the extracellular matrix under flow rates of 30 and 50 μL/min increased 2.2-fold and 4.4-fold, respectively, compared to the static culture conditions. 

### 3.3. Scanning Electron Microscopy (SEM) Images of the Collagen Substrate

**Figure 4:**
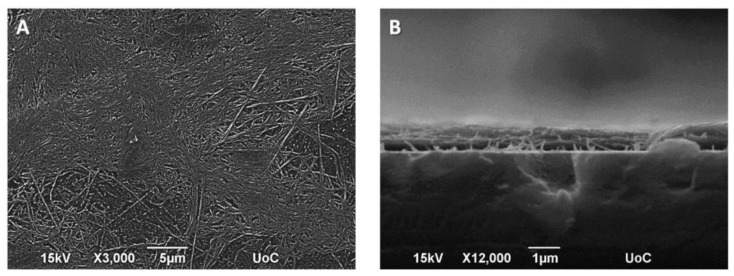
SEM images showing the morphology of fibrous collagen networks (**A**); SEM image of a sample cross-section showing the fibrous collagen scaffold prepared on a glass substrate (**B**).

Figure 4A depicts the SEM image of a network of thick collagen fibers prepared at 4 °C for 48 h, at 3000× magnification. The thickness of the fibrous collagen scaffold was determined by SEM after the creation of a sample cross-section analysis and was found to be around 700 nm (Figure 4B). 

### 3.4. Rheological Measurements of the Collagen Substrate

**Figure 5:**
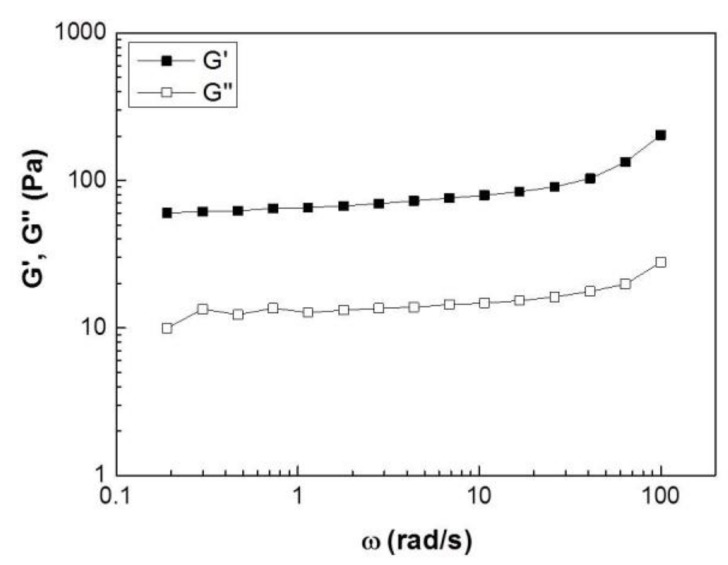
Linear viscoelastic response measured by a Dynamic frequency sweep test for a 3 mg/mL collagen hydrogel at 37 °C. The elastic modulus, G’, is represented by solid symbols and the viscous modulus, G’’, by open ones.

Dynamic frequency sweep experiments were performed to determine linear viscoelastic response measuring the elastic (or storage), G’, and viscous (or loss), G’’, moduli. The tests were performed in the linear regime with a strain amplitude of 0.5% found to be in the linear regime by dynamic strain sweeps performed at a frequency of 1 rad/s. Figure 5 shows such data for a 3 mg/mL collagen gel at 37 °C. The spectra reveal a typical viscoelastic response of a soft solid with an elastic modulus, G’, which exhibits a weak increase in the frequency range investigated, and a viscous modulus, G’’, that is an order of magnitude lower than G’’. Therefore, over the entire range of applied frequencies, the gel sample exhibits a soft solid-like behavior with no sign of a slow relaxation mode at long times (up to 1/ω~30 s) that would suggest that the sample would flow ( exhibit a liquid-like behavior). 

### 3.5. Cell Seeding of Collagen Substrates with MC3T3-E1 Cells

**Figure 6:**
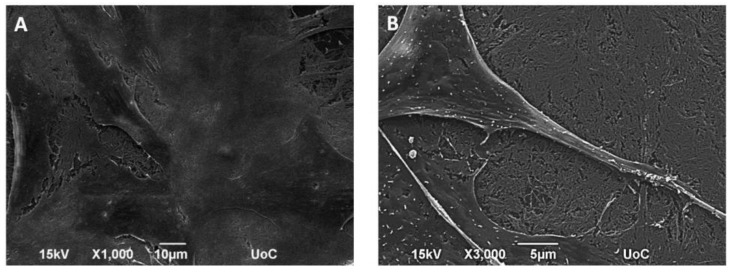
SEM images show the morphology of MC3T3-E1 cells after 1 day of culture spreading onto fibrous collagen; images with different magnifications, 1000× (**A**) and 3000× (**B**) indicate a fully flattened pre-osteoblastic cell morphology and cell protrusions, respectively.

The cell–biomaterial interactions are depicted in Figure 6, after one day in culture, at 1000× (Figure 6A) and 3000× (Figure 6B) magnification. On the first day, most cells exhibited a branched shape and flattened morphology with long cellular extensions that signal good adhesion and growth of the cells on the collagen fibers [29]. 

### 3.6. Pre-Osteoblastic Cell Morphology and Orientation on Collagen Substrates under Static and Flow Conditions

**Figure 7:**
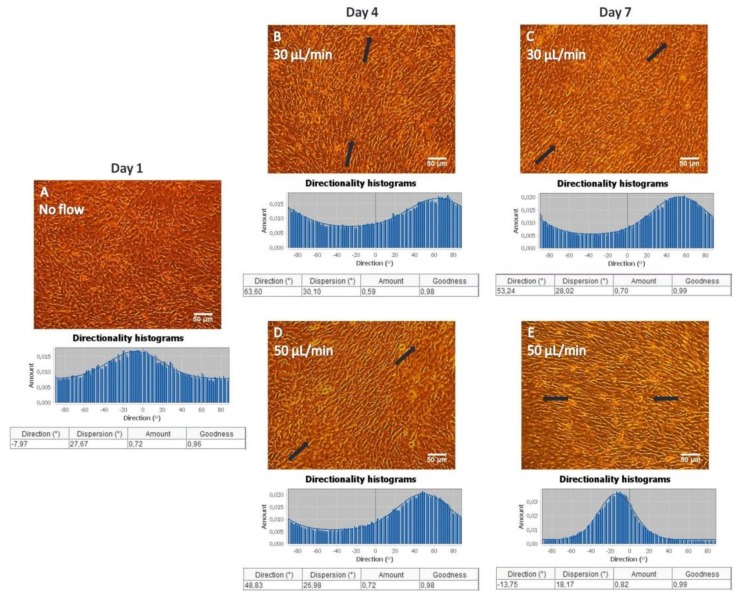
Morphology of MC3T3-E1 cells inside the flow perfusion culture system using fibrous collagen substrates under static conditions (**A**), and under flow conditions applying 30 (**B**,**C**), and 50 μL/min (**D**,**E**) using optical microscopy (ten-fold magnification, the scale bar represents 50 μm). The black arrows represent the direction of the flow. Directionality histograms and tables with the statistics generated by means of the Fiji ImageJ plug-in “Directionality” [25] are presented directly under the optical microscopy images (**A**–**E**).

Similar to gelatin substrates, MC3T3-E1 cells adhered strongly and proliferated on collagen substrates both under static (Figure 7A) and flow conditions, with flow rates of 30 (Figure 7B,C) and 50 (Figure 7D,E) μL/min, as observed in Figure 7. An orientation of the pre-osteoblastic cells along the direction of the flow was obtained when applying the flow rates of 30 (Figure 7B,C) and 50 (Figure 7D,E) μL/min, whereas a random orientation was observed under static conditions (Figure 7A). This is also demonstrated by the directionality histograms confirming that the amount is concentrated each time at the direction of flow [~60° (Figure 7B), ~50° (Figure 7C,D), ~−10° (Figure 7E)] compared to the static culture (Figure 7A). The black arrows represent the direction of the flow.

### 3.7. Proliferation of MC3T3-E1 Cells on Collagen Substrate under Static and Flow Conditions

**Figure 8:**
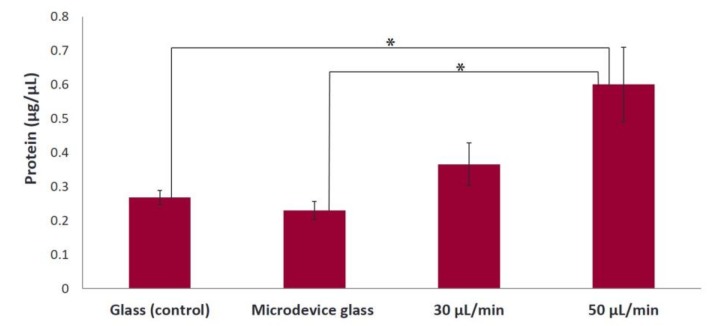
Amount of cells expressed as total protein concentration after 7 days of culture on fibrous collagen substrates under static (glass and microdevice glass) and flow conditions (flow rates of 30 and 50 μL/min). A *p value of < 0.05 was considered significant.

The results of the determination of total protein concentration, on fibrous collagen substrates, on day 7 are depicted in Figure 8. A significant difference in the proliferative cell behavior is observed under dynamic conditions, with a flow rate of 50 μL/min with a 2.4-fold cell proliferation increase compared to under static conditions (glass and microdevice glass) (*p < 0.05). The cell proliferation was increased under the condition with a flow rate of 30 μL/min compared to the static culture, without significant differences being observed. 

### 3.8. Alkaline Phosphatase Activity of MC3T3-E1 Cells on Collagen Substrate under Static and Flow Conditions

**Figure 9:**
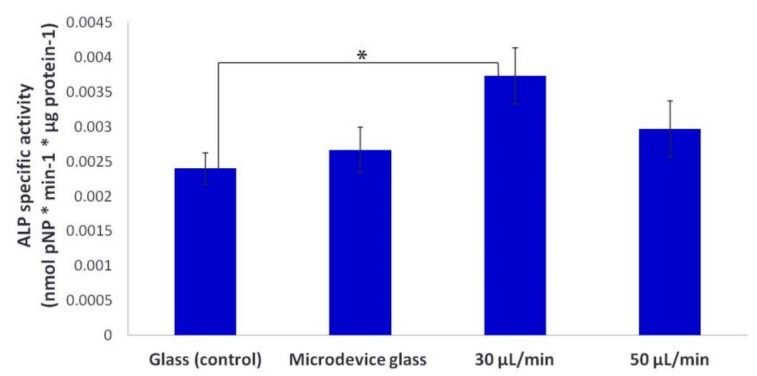
Normalized ALP activity of MC3T3-E1 cells on fibrous collagen substrates after 7 days of culture under static (glass and microdevice glass) and flow conditions (flow rates of 30 and 50 μL/min) in the presence of osteogenic medium. A *p value of < 0.05 was considered significant.

The ALP activity was determined on day 7 in culture and normalized with the total protein concentration. We observed that the normalized ALP activity on fibrous collagen substrates was with a 1.6-fold increase significantly higher (*p < 0.05) under the 30 μL/min flow rate condition compared to the static condition (glass). The ALP activity was higher under the 50 μL/min flow rate condition compared to the static conditions; however, it was not significantly different (Figure 9). 

## 4. Discussion

Bone remodeling occurs due to the fact that bone cells respond to mechanical stimulations. One of the main mechanical stimulations on bone cells, in vivo, is the interstitial fluid flow stress in the lacunae-canaliculi network [30]. However, on conventional static cultures, this crucial parameter of fluid flow stress is neglected. Microfluidic cell culture provides an extra degree of control over cultured cells by delivering not only chemical but also mechanical signals, through fluid flow, exerting a shear stress on adherent cells. Laminar flow resembles the physiological fluid flow in the body, facilitates mass transport of solutes, and supplies consistent nutrient delivery and effective waste removal, similar to an in vivo environment. One of the advantages that microfluidics bring to cell culture is the continuous flow of fresh media within the culture system [12,31].

In the present study, we first set up a new precisely flow-controlled microfluidic system and investigated the potential of pre-osteoblastic cells on gelatin as test substrate to control directional growth, as well as their ability to form extracellular matrix, by applying flow rates and consequently velocities similar to those of the small arteries in the bloodstream. Once we received promising results on the response of MC3T3-E1 cells on gelatin substrates, we focused our investigation on the biological response of cells on fibrous collagen scaffolds as substrates for cells in the microfluidic chamber. For this, we characterized the collagen substrates morphologically and rheologically, and evaluated the orientation, proliferation and osteogenic differentiation capacity of MC3T3-E1 cells cultured on them.

Under both flow rates of 30 and 50 μL/min, MC3T3-E1 cells appeared to be oriented along the direction of flow, whereas cells depicted a random orientation under static conditions on gelatin substrates as confirmed by the directionality histograms (Figure 2). In bone cells, the cytoskeleton is considered to be the major factor that influences the cellular morphology and biomechanical response [32]. Cellular morphology determines cellular function and is related to the organization of cytoskeletal components. The cytoskeleton is a fundamental structure that contains a network of microfilaments and microtubules. When the cytoskeleton is elongated, there are more docking and activation sites for focal adhesions, which act as mechanical linkage to the extracellular matrix. Mechanical stimulation affects the cytoskeletal organization producing different cellular responses. In vivo, the magnitude of the fluid flow stress in bone tissue is estimated to be between 8 and 30 dynes/cm^2^ [33]. It has been reported that osteoblasts have been used to investigate the morphological and functional responses to fluid shear stresses in vitro [13,14]. The values of the shear forces reported being stimulatory for pre-osteoblastic cells cultured in two-dimensional flow chambers for short periods are higher than 2 dynes/cm^2^ [34]. In this study, the approximate fluid shear forces experienced by the cells were calculated to be in the range of 0.3–0.5 dynes/cm^2^, and although they are lower than the reported values, the cells were able to respond to these fluid shear forces both morphologically, as shown in Figure 2, and functionally, as presented in Figure 3. The collagen produced in the extracellular matrix under flow rates of 30 and 50 μL/min using gelatin substrates increased 2.2-fold and 4.4-fold, respectively, compared to static conditions (Figure 3). Possible mechanisms for the increased collagen production in the extracellular matrix include the exposure of the seeded cells to fluid shear, which induces mechanical stimulation and the decrease of nutrient transport limitations experienced by the cells cultured under static conditions [7,23].

The gel elasticity directly affects cell spreading, migration and contraction, and organizes extracellular structures such as focal adhesions, as well as the cell proliferation and differentiation [16,17]. Thus, we investigated the rheological properties of collagen hydrogels to better understand the mechanical environment of cells. The rheological analysis showed a typical weak solid-like viscoelastic behavior, with the values of storage modulus (G’) being larger than loss modulus (G”) over the applied frequencies (Figure 5). 

Similar to gelatin substrates, an orientation of MC3T3-E1 cells on collagen matrices along the direction of the flow was observed when applying the flow rates of 30 and 50 μL/min, whereas a random orientation was observed in static conditions, which was also evidenced by the directionality histograms (Figure 7). Moreover, we found that the cell proliferation increased 2.4-fold under a flow rate of 50 μL/min compared to static conditions (Figure 8). The increased proliferation under flow conditions is in agreement with previous studies [6,9,27], showing that the use of dynamic cultures leads to increased cell proliferation. A possible explanation is the improved supply of nutrients and mass transport of oxygen within the collagen fibers. Indeed, in static cultures, there are mass transport limitations to the interior of the collagen fibers, which are absent under flow conditions. In addition, the continuous flow could enhance the effective waste removal of cell metabolic products from the interior of the fibrous network. The stimulatory effect of fluid flow on the proliferation of pre-osteoblastic cells seeded on collagen fibers could be attributed to the fluid shear forces that pre-osteoblasts experience, as well as the enhanced nutrients transport provided by the continuous perfusion of the medium. Thus, the collagen fibers were able to support increased cell growth under flow conditions as evidenced by the higher cell proliferation compared to static conditions.

Moreover, we observed that the ALP activity of MC3T3-E1 cells on collagen matrices under the flow rate of 30 μL/min was significantly higher compared to static conditions (Figure 9), which is in line with previous reports on bone marrow stromal cells [8,27,35]. However, the ALP activity under the flow rate of 50 μL/min was slightly higher than that under static cultures. This can be attributed to the enhanced proliferation of the cells (as presented in Figure 8) as a dominating process against the differentiation during the experimental period of seven days. Specifically, the cell growth over time and the expression of the osteoblastic phenotype have been shown to occur in three periods: (i) the first period marked with a strong proliferation and formation of extracellular collagenous matrix, (ii) a phase of matrix maturation featured with a decrease of proliferation and increase of the ALP expression, and (iii) a mineralization phase with a further decrease of proliferation, and of the ALP activity, and formation of mineralized extracellular matrix [36]. It is not clear whether the enhanced proliferation and differentiation of pre-osteoblastic cells on the fibrous collagen substrates is solely due to the improved nutrient supply within the collagen fibers, or due to the stimulation of the seeded cells through their exposure to fluid shear forces. Probably both events are involved in controlling cell proliferation and differentiation as previously reported [27]. Although the fluid shear forces experienced by the pre-osteoblastic cells within the range of 0.3–0.5 dynes/cm^2^ are lower that the values reported as stimulatory for cells cultured into two-dimensional (2D) flow chambers for short time periods [34], our findings on the proliferative behavior and the ALP activity indicate that the applied shear forces enhanced the proliferation and osteogenic differentiation of pre-osteoblasts in long-term cultures. 

Our findings demonstrate that precisely flow-controlled microfluidic cell culture is important for directing cellular activities in a controlled cell microenvironment, and highlight the potential of this system to control autologous graft formation for accelerated bone reconstruction.

## Figures and Tables

**Table 1:** Values for mean velocity ($\bar{u}$), diameter (*d*) and flow rate (*Q* = ($\bar{u}$π$d^{2}$)/4) of the vessels in the blood circulation [22,23].

Blood Circulation			
Vessel	$\bar{u}$ (m/s)	*d* (mm)	*Q* (μL/min)
Aorta	0.4	25	11.8 × 10^6^
Arteries	0.45	4	3 × 10^5^
Arterioles	0.05	0.05	5.9
Capillaries	0.001	0.008	0.003
Venules	0.002	0.02	0.038
Veins	0.1	5	12 × 10^4^
Vena cava	0.38	30	16.1 × 10^6^

**Table 2:** Values for flow rate (*Q*), tubing diameter (*d*) and mean velocity (($\bar{u}$ = (4*Q*)/(π$d^{2}$)) in the microfluidic system [23].

Microfluidic System		
*Q* (μL/min)	*d* (mm)	$\bar{u}$ (m/s)
30	0.5	0.0026
50	0.5	0.0042

**Table 3:** Values for flow rate (*Q*) and shear stress (*σ*) in the microfluidic system.

Microfluidic System	
*Q* (μL/min)	*σ* (dynes/cm^2^)/(N/m^2^)
30	0.3/0.03
50	0.5/0.05
